# Scent of a Symbiont: The Personalized Genetic Relationships of Rhizobium—Plant Interaction

**DOI:** 10.3390/ijms23063358

**Published:** 2022-03-20

**Authors:** Lisa Cangioli, Francesca Vaccaro, Margherita Fini, Alessio Mengoni, Camilla Fagorzi

**Affiliations:** Department of Biology, University of Florence, Via Madonna del Piano 6, 50019 Sesto Fiorentino, Italy; lisa.cangioli@unifi.it (L.C.); francesca.vaccaro@unifi.it (F.V.); margherita.fini@stud.unifi.it (M.F.); camilla.fagorzi@unifi.it (C.F.)

**Keywords:** rhizobium genomics, symbiotic interaction, nitrogen fixation, sustainable agriculture

## Abstract

Many molecular signals are exchanged between rhizobia and host legume plants, some of which are crucial for symbiosis to take place, while others are modifiers of the interaction, which have great importance in the competition with the soil microbiota and in the genotype-specific perception of host plants. Here, we review recent findings on strain-specific and host genotype-specific interactions between rhizobia and legumes, discussing the molecular actors (genes, gene products and metabolites) which play a role in the establishment of symbiosis, and highlighting the need for research including the other components of the soil (micro)biota, which could be crucial in developing rational-based strategies for bioinoculants and synthetic communities’ assemblage.

## 1. Introduction

Plants, like other multicellular eukaryotes, live in close association with many other organisms, and they often establish mutualistic relationships with them which, in turn, shape their phenotype. Indeed, many host plant traits are now recognized as being influenced by host-associated organisms, especially microorganisms, either prokaryotic and eukaryotic [1]. The plant phenotype is a result of multiple-level interactions, starting with the host genotype, and including physicochemical environmental factors, but also, more importantly, the microbiome composition, behavior and function [2,3]. There is a large debate on how to consider the heritability of microbe-related traits [2]. It has been suggested that phenotypic variation should be considered as the sum of environmental variance and of additive and nonadditive contributions. These contributions derive from the genotype of the host and of the mutualistic microbes (including their mobile genetic elements, which largely vary among strains) and from the intergenomic epistasis between hosts and microbes [4].

Disentangling these multi-layered, interconnected interactions is complicated. Research can be performed on natural populations by applying statistical methods to disentangle the contribution of the variables [5], or by using model systems in controlled conditions [6]. However, the extreme diversity (genetic and functional) of the native host-associated microbiome limits the possibility of understanding the molecular and genetic factors of the interactions, although relevant works addressing the role of plant genotypes and single genes have been conducted [7,8,9,10].

The rhizobia–legume plant mutualistic interaction offers a simplified, though still complex, system to address questions on the heritability of microbe-related traits delving deep into deciphering the genetic determinants and the molecular communication in the establishment of successful associations. Rhizobia are ubiquitous soil bacteria, belonging to the Alpha and Beta Proteobacteria classes, which colonize the rhizosphere and endosphere of different plant species and form symbiotic associations with leguminous plants [11]. The symbiotic association typically starts with the perception of plant-derived flavonoids that bind the bacterial NodD protein, which, in turn, activates the transcription of *nod* genes, resulting in the production of lipochitooligosaccharides (LCOs) termed Nod factors (NFs). NFs are perceived by the host legume and trigger the division of inner plant cortical cells, resulting in the formation of nodule primordia, and root hair curling which induces infection thread formation [12]. This process facilitates the accommodation of rhizobia inside the plant cell, where they differentiate into bacteroids and start fixing atmospheric nitrogen [12]. Metabolic rewiring and the stress response play crucial roles in symbiotic establishment [13,14], since bacteroids must cope with osmotic stress, changes in pH, reduced oxygen tension [14] and plant peptides. Plant peptides are becoming increasingly relevant since they induce and maintain growth inhibition and can modify host compatibility [15,16,17,18,19]. Related to nodule growth and correlated with bacteroid differentiation, nodules can be found in determinate and indeterminate forms. Determinate nodules are found in tropical and subtropical legumes such as *Lotus japonicus*, soybean (*Glycine max*), common bean (*Phaseolus vulgaris*) and cowpea (*Vigna unguiculata*). Temperate legumes tend to form indeterminate, and one of the most studied models is the legume *Medicago truncatula* and its symbiotic rhizobium *Sinorhizobium meliloti*. Indeed, many of the genes required by the plant and the symbiotic bacterium for establishing a successful symbiosis have been discovered in this model, and genetic tools that enable testing hypotheses have been developed [20,21]. Therefore, while this review aims to be general, most of the research data and examples provided are related to indeterminate nodules and to the model system *M. truncatula*/*S. meliloti*. This system offers excellent working models to molecularly understand host–microbe relationships and host genotype–microbial genotype interactions [20].

## 2. Evidence of Genotypic Signature in the Rhizobium—Plant Partner Choice

Several population genetics investigations on rhizobial symbionts have been performed on many species, spanning from soybean symbionts to fava bean and alfalfa symbionts, initially with PCR-based approaches, then more recently with genome sequencing (see, for instance, [22,23,24,25,26,27,28,29,30]). Since the first works, it was clear that strains isolated from nodules of different host plant genotypes (e.g., varieties, or closely related species) cluster, to some extent, into different groups. More than 20 years ago, a long-term experiment where physically separated nodulating populations were followed over the years on the same plants showed increasing relationships with the host plant genotypes, though random genetic drift was also an important factor shaping the rhizobial population structure [22]. This long-term evolution experiment with combinations of different cultivars of *Medicago sativa* and soils allowed monitoring the genetic diversity of *Sinorhizobium meliloti* strains isolated from nodules formed over the years by the same single plant. The results showed that the genetic structure of the symbiotic rhizobial population was influenced, to different extents, by factors such as the soil type, plant cultivar, individual plants within the same cultivar and time. At the beginning of the experiment, the genetic variability of *S. meliloti* strains was mainly related to the soil and the host plant cultivar, while over the years, the effects of these two factors were no more significant, and the individual plant played the most important role [22]. Since the cultivars used also showed a relatively high genetic polymorphism [31], the individual plant effect retrieved could be attributed to either random genetic drift of the nodulating rhizobial populations or plant genotype selection.

Several pieces of evidence accumulated later confirmed that the symbiotic phenotype, including the amount of the bacteroid-differentiated population inside root nodules, is influenced by both the rhizobial strain and plant line [19]. Recently, the host nodule-specific PLAT domain (NPD) gene family, affecting late-stage nodule development and host biomass [30], as well as plant-nodule-specific Cys-rich peptides (NCRs), has been shown to have strain-specific effects on rhizobial fitness in competition [32]. The NPD gene family is composed of five genes that are expressed during late nodule development. NDP knockout mutants showed that the disruption of NDP genes led to changes in symbiotic phenotypes (using *S. meliloti* 1021 as a rhizobial symbiont), including plant size, number of mature (pink) nodules, time of nodule senescence and the area of the fixation zone in nodules [30]. Interestingly, plants inoculated with a mixed population of rhizobial strains and with single strains showed phenotypic differences, suggesting that the effects of mutating NDP genes are rhizobial strain-specific [30]. Another important factor which can be related to partner choice is plant-encoded nodule cysteine-rich peptides (NCR peptides) involved in the formation of indeterminate nodules. NCR genes are exclusively expressed in infected nodule cells, demonstrating their relevant role as players in the regulation of symbiotic development. We discuss the relevance of NCR peptides later in this paper; however, here, it is important to point out their role in partner choice. The rhizobial symbiont in fact seems to be protected against the antibacterial activity of NCR peptides by a specific gene, *bacA* [15,33]. As a proof of this hypothesis, heterologous complementation of BacA from different rhizobial species produced host species-dependent symbiotic outcomes [34]. Moreover, an evolutionary scenario of host–symbiont coevolution with a rapid diversification of the NCR and *bacA* genes in legumes has been reported [33,34]. This evidence may suggest that allelic forms of the *bacA* gene may be involved in strain x host genotype specificities.

## 3. Fitness Alignment between Rhizobium and Host Plant

Symbiotic interactions between nitrogen-fixing rhizobia and host plants are a clear example of classical mutualism, where the increase in the population size of one partner causes an increase in its partner’s population size. Rhizobium inoculation has a beneficial effect on legume growth, and the growth of a compatible host increases the relative abundance of rhizobia in the soil [35]. However, conflicts in symbiosis may arise in terms of conflicts of interest between the symbiont and the host and fitness conflict in the use of resources (cheating). A meta-analysis showed a widespread alignment between the host’s and the symbiont’s fitness [36]. Mutant studies have shown that a large number of mutations that are deleterious to the host are also deleterious to the symbiont’s competitive ability, and, conversely, that beneficial mutations for the symbiont increase the host’s growth. Cheater mutations, increasing rhizobium fitness at the host’s expense, are rare. In fact, a trade-off between rhizobium fitness, use of resources and plant fitness exists. An integrated metabolic model reconstruction between the host plant *M. truncatula* and the symbiotic rhizobium *S. meliloti* has highlighted the fitness conflicts in the use of resources [37]. Here, increasing nodule biomass (therefore allowing more rhizobial cells to grow) initially increases host biomass, but after, a decrease in plant biomass gradually occurs. Considering nitrogen fixation efficiencies, simulations in the metabolic model indicated a linear relationship between nitrogen fixation and biomass production until an optimal level, but then excessive nitrogen fixation quickly resulted in a drop in plant biomass due to insufficient energy to support both the nitrogen fixation and ATP production costs. This pareto frontier between the nitrogen fixation efficiency and the rate of plant biomass production probably contributes to reducing cheating mutations, therefore aligning the symbiont and host fitness. However, we should consider that most of the studies measured plant fitness as plant growth/biomass production and did not directly measure reproductive capacity (e.g., seed production). Experiments directly assessing plant fitness would be needed to evaluate the alignment in fitness between the rhizobium and host plant in more depth. Moreover, directly measuring rhizobial cells released from nodules would also be relevant [38], in order to better understand the strategies of determinate and indeterminate nodule types, the latter harboring terminally differentiated bacteroids [36,39]. Moreover, most of the performed studies included single rhizobial strains, but competition between rhizobium strains in the formation of nodules exists, as well as evidence of variable levels of mutualisms in natural symbiont populations [40,41,42,43]. These give rise to the need for additional control over the mutualistic ability of the symbiont [44,45,46]. Experimental strategies, such as cycles of selection of a symbiont population, transposon insertion libraries, the use of massive genomic sequencing [47,48] and genome-wide association studies [41], allow shedding light on the genetic determinants for competition. These studies have shown an incredible number of putative loci in the symbiont’s genome, which can be related to competition and fitness in the indeterminate nodule colonization. Clarifying the importance of such loci in terms of their contribution to the host’s fitness is compulsory, especially in relation to the use of rhizobia as inoculants in agriculture with the aim to increase legume crop yield [49]. Strains harboring large-scale genomic manipulations, such as those having a minimal symbiotic genome [50,51] and large genome manipulations [52], in addition to other more classical genetic approaches [20], would be useful to experimentally test predictions based on genome analyses and computational models.

## 4. Detecting Genotype × Genotype Interaction in Nodule Colonization

During the molecular dialog between legumes and rhizobia, several aspects come into play when the partner choice is to be made [53]. Phenotypic studies in legumes and rhizobia have shown that even within a species, symbionts are not all equivalent in the fitness benefits they confer to their hosts, and conversely, hosts differ in the fitness benefits they confer upon symbionts [54,55]. Sanctions are one of the possible strategies the host can choose to control the resource environment of their symbiont [56]. Legumes select for more cooperative rhizobia by imposing sanctions based on the amount of nitrogen fixed once bacteria are established inside nodules: effective symbionts are favored through the sanction of noneffective symbionts [57]. Several works have been conducted that attempt to understand the basis of sanctions and partner choice and the genetic determinants underlying preferences in host and symbiont partnerships [14,42,44,57,58,59]. However, only a few genes have been clearly identified as related to host preference. A classical element related to host preference is the *nod* genotype of the rhizobium, related to NF production. NFs are characterized by oligosaccharide chains of different lengths, distinct fatty acids and several substitutions, which determine the host range of the rhizobium, either among species or among strains of the same species (symbiovars) [60]. The *nod* genotype of rhizobia includes a set of genes encoding the enzymes that synthesize LCOs, whose basic structure is synthetized by proteins encoded by the *nodABC* genes [61]. The *nodC* gene is one of the most relevant variations in the LCO structure and a key determinant of species-specific recognition [62]. Interestingly, this recognition can be poorly specific, with a legume host being nodulated by different species/strains of rhizobia, showing extensive polymorphism in the *nodC* gene (e.g., *Glycine max*, *Lotus* spp., *Phaseolus vulgaris, Sophora flavescens*, *Arachis hypogaea*), or highly specific (e.g., *Cicer arietinum*, *Astragalus sinicus*, *Trifolium* spp.), requiring the presence of specific rhizobial species, with a highly conserved and distinct *nodC* gene sequence [62].

Alongside the now well-elucidated set of genes and functions related to the core symbiotic equipment of rhizobia (such as *nod* genes involved in Nod factor biosynthesis and flavonoid perception [63]), much remains unknown about the modifiers/accessory genes required to optimize the interaction. Genome-wide analyses of plant-associated microbes have found enrichment in genes related to carbohydrate metabolism, the type III secretion system, phytohormone production and phosphorus solubilization [8,64,65]. However, concerning the intraspecific selectivity of legume–rhizobia symbiosis, interaction with plant innate immunity seems to play an important role [66,67,68]. The interaction with plant immunity relies on various aspects of rhizobia (e.g., secretion systems, exopolysaccharides, proteolysis of plant antimicrobial peptides). Different rhizobial secretion systems (type III, type IV and type VI) influence rhizobial host specificity and the number of nodules, possibly modulating the transport of effector proteins into host cells [69]. Additionally, exopolysaccharides have been linked to infection and colonization processes by supporting bacterial attachment to the root surface. In rhizobia, secreted exopolysaccharides contribute to building a biofilm that increases nutrient absorption and enhances communication between plants and bacteria [70]. Early studies showed that mutation of genes related to the synthesis of exopolysaccharides (*exo* genes) in *S. meliloti* resulted in ineffective nodules on alfalfa, containing no bacteroids [71].

In general, the more studies are performed, especially coupling genome-wide mutant libraries with testing of symbiotic competitiveness, the more genes are found. These genes are involved in a plethora of functions, including metabolic pathways, transporters, chemotaxis and motility, from systems spanning from the *Bradyrhizobium diazoefficiens* host range to *S. meliloti* [48,72,73]. In addition to simple gene presence/absence on the rhizobial symbiont, recent genomic and transcriptomic studies have stirred the attention on genotype-by-genotype (G × G) interactions in symbiotic outcomes [41,49,52,74,75]. Experiments carried out on *M. truncatula* and *S. meliloti* strains demonstrated that the ability to enter plant nodules increased rhizobium fitness, and plants reward more cooperative rhizobium strains by preferentially forming nodules with them, but the rewarded strains differ among plant genotypes [45]. This evidence highlights how the outcomes of rhizobium fitness (hence their evolution) depend on the genetic composition of the plant populations they interact with.

**Figure 1:**
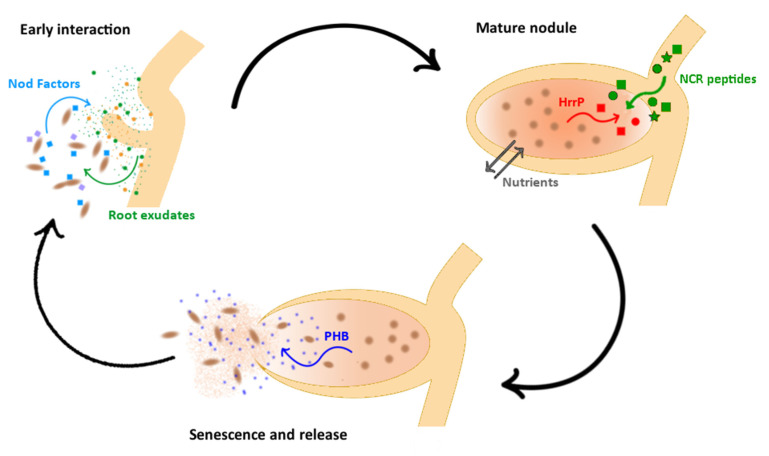
Genotype × genotype (G × G) interactions in the nitrogen-fixing root nodule symbiosis. A schematic example of three phases of symbiosis, namely, early interaction with roots, mature nodules and release of bacteria from dehiscent nodules, is shown. The key molecular actors identified along the phases are reported.

Indeed, during the partner recognition nodule formation process, G × G interactions could act at different levels. In the early nodule developmental stage, i.e., during the reciprocal perception of the partners, symbiotic specificity is based on the rhizobial perception of root exudates presenting host-secreted flavonoids and the subsequent host perception of rhizobial Nod factors [76]. Plant exudates, key determinants of the rhizosphere microbiome structure, vary among plant species and different varieties of the same plant species [77]. By maintaining a broad repertoire of transporters, rhizosphere bacteria, including rhizobia, might efficiently capture resources from different types of hosts and successfully compete during plant colonization [64]. In *S. meliloti*, the pSymB chromid harbors many genes for membrane transporters, having roles in rhizosphere colonization and utilization of nutrients present in root exudates [78,79]. Deletion of pSymB has been shown to impair rhizosphere colonization [78,80]. Interestingly, the transcriptional patterns induced in *S. meliloti* by root exudates from different varieties of alfalfa changed according to the variety [55]. Moreover, different strains of *S. meliloti* responded differently to the same variety, clearly showing a G × G-related pattern of transcriptional response changes [55]. In the same work, a goodness-of-fit model (nested likelihood ratio test) indicated that nearly up to 30% of the total differentially expressed genes were explained by strain x variety interaction. Similar results were also obtained from the analysis of nodule transcriptomes (hence the mature phase of symbiosis) [54]. Among the retrieved genes, the main functions were cellular energy production pathways and processes playing roles in the exchange of nitrogen and carbohydrate utilization. Still concerning the nodule environment, during the shift from non-differentiated, reproductively capable rhizobia to the no longer reproductively competent and differentiated bacteroids, differential expression of host genes may represent mechanisms used by hosts to control differentiation and nitrogen fixation in rhizobia [81]. In this context, a group of genes encoding for nodule cysteine-rich peptides (NCR peptides) triggered interest for their possible role in modulating the outcome of symbiosis and as candidates for determining post-infection host–rhizobia specificity (Figure 1). Legume NCR peptides were initially discovered as antimicrobial molecules limiting the reproductive potential of endophytic rhizobia under sub-lethal concentrations [82,83]. However, they have been recently reconsidered as decisive for the bacteria to adapt to the intracellular environment, therefore being needed for a successful symbiosis [84,85]. On the other hand, some rhizobia are not challenged by NCR peptides during symbiosis, as NCR peptides are not present in their respective host legumes such as soybean and *Lotus* spp. [86]. This may be due to the disadvantage of legumes to impose terminal differentiation on bacteroids in legume species lacking persistent infection threads [86]. However, a comprehensive interpretation of the reason NCR peptides are essential for symbiotic nitrogen fixation in some legume species and not in others is still lacking [86]. Screening of NCR peptide genes from several *M. truncatula* accessions reported significant expression and sequence variation, suggesting they can be involved in symbiotic phenotypic variation among accessions [87]. Indeed, NCR peptides are responsible for many of the metabolic and morphological changes observed in bacteroids in nodules [33]. In a classical Red Queen evolutionary scenario [88], a rhizobial-encoded peptidase, termed as host range restriction peptidase (HrrP), was shown to allow the modulation of the *S. meliloti* host range toward different *Medicago* species by specific proteolysis of host-derived NCR peptides [89] (Figure 1). Experiments of transposon mutagenesis followed by high-throughput sequencing (Tn-seq) have identified genes, in addition to HrrP, that increase or decrease bacterial competitiveness during exposure to the NCR peptides in *S. meliloti*. These genes relate to several cellular processes such as polysaccharide biosynthesis, membrane proteins, peptidoglycan effector proteins, transcriptional regulators and ribosome-associated factors. It is interesting to note that for a single component of nodule colonization, such as the NCR sensitivity, as well as for the early interaction with roots, a panoply of genes is involved.

Among the sanctions the plant can induce on nodules, senescence related to ethylene production has also been discussed. Strains of rhizobia expressing the 1-aminocyclopropane-1-carboxylate (ACC) deaminase enzyme, a possible scavenger of the ethylene precursor ACC, have been shown to have, in some cases, advantages for nodule colonization [40], though in *S. meliloti*, the gene *acdS*, encoding for ACC deaminase, also seems to be related to rhizosphere colonization and the use of unusual nitrogen sources [90]. Manipulation of ethylene by rhizobia is also due to rhizobitoxine [91]. Strains producing rhizobitoxine are more competitive over nonproducing strains and also accumulate more carbon resources, such as poly-β-hydroxybutyrate (PHB) [92]. PHB accumulation inside bacteroids is claimed to be a measure of rhizobial fitness, once the dehiscent (determinate) nodules release the bacteria [38] (Figure 1). PHB stabilizes the cellular redox condition and provides a carbon source for bacteroids/undifferentiated rhizobia when released from the nodule to the soil [93,94]. While some strains (e.g., *Sinorhizobium* sp. NGR234, *B. elkani* USDA 76, *Sinorhizobium meliloti* CCBAU01199) produce a large amount of PHB, others (e.g., *M. loti* NZP2213, *B. arachidis* CCBAU 051107, *M. septentrionale* CCBAU03399) produce only a few PHB granules [95]. It has been shown that a single rhizobial population may display bet-edging on PHB, with two subpopulations, one with low PHB which has greater competitiveness for resources, and another with high PHB which can survive until a legume host is next available [93,96]. However, the role of genetic variability in ethylene modulation and PHB production in connection with plant metabolism (a G × G interaction) has not been addressed yet.

We can argue that many molecular actors in modulating G × G interaction in symbiosis are still unknown, and we need a more comprehensive approach, using systems biology simulation and multi-omics data interpretation, combined with careful phenotypic analyses, to fully disclose the network of relationships among rhizobial and plant genes.

## 5. Molecular Tasting in the Rhizosphere

Bacterial root colonization (on the rhizoplane and in the endosphere) is stimulated by the panoply of molecules released by the plant root tissues, either as volatile or soluble molecules. Concerning volatile molecules (often referred to as volatile organic compounds, VOCs), relatively few are known in relation to direct bacterial chemoattraction. Only recently has a role of volatile compounds in recruiting beneficial environmental bacteria, for instance, under stress conditions (the “crying-for-help” strategy [97]) been demonstrated [98,99,100]. However, no specific role of plant-emitted VOCs has been assigned to the symbiosis with rhizobia. On the contrary, for rhizobium-emitted VOCs, there is evidence that they may have a role in the promotion of non-host plant growth and in the increase in iron uptake mechanisms, rhizosphere acidification and increased root ferric reductase in host legumes [101]. However, most of the research and data are on soluble molecules, emitted by roots and microbes. Concerning the microbial contribution, very detailed models of bacterial population density control and adhesion to plant roots via biofilm formation are available [102]. Quorum sensing as microbe–microbe signaling is a well-known phenomenon mediated by molecules such as N-acyl homoserine lactones (AHLs) [102]. In rhizobia, quorum sensing systems are related to exopolysaccharide production and early root colonization [103], and to symbiotic plasmid transfer in some species such as *R. leguminosarum* bv. *viciae* (pRL1JI) and *Sinorhizobium fredii* NGR234 (pNGR234) [104]. Interestingly, several compounds from the plant side (present in root exudates) are agonists of AHL signaling [105], indicating that host plants can manipulate the bacterial behavior, possibly favoring the expression of bacterial genes that are beneficial to them. Indeed, root exudates are a very complex mixture of substances, which vary depending on the plant genotype, plant growth stage and substrate, dramatically affecting root microbiota [106,107,108]. Legume root exudates include sugars, organic acids and amino acids in large amounts, but also molecules such as flavones and flavonoids, well-known inducers of nodulation signaling [109,110,111]. These compounds elicit various functions in the rhizobia present in the rhizosphere, spanning from metabolic modules [80] to transcriptional changes in genes related to chemotaxis and transport [112], and also mirroring competitive abilities [113]. In a model of G × G interaction between alfalfa (*Medicago sativa*) varieties and strains of the rhizobium *S. meliloti* [74], different plant varieties affected the transcription of different sets of rhizobial genes, including those involved in quorum sensing systems and motility. Since the composition of root exudates among alfalfa varieties mainly differs in the content of amino acids, such as N-acetyl-L-leucine, tryptophan, cytosine, 3,5-dihydroxyphenylglycine and some dipeptides [74], we can hypothesize that some of these molecules may play a signaling role with respect to symbiotic rhizobia. In line with hypotheses on the presence of molecules which can differentially modulate rhizobia gene expression and behavior in root exudates, there is evidence that root exudates from non-host plant species (such as wheat) may synergistically act to promote rhizobia–host legume symbiotic interaction [114].

Of course, the most well-known signaling molecules present in legume root exudates are flavonoids such as luteolin, quercetin and apigenin. Flavonoids modulate the host specificity of symbiotic rhizobia [115], selectively binding and activating the protein NodD, which transcriptionally regulates the so-called nodulation (*nod*) genes. These are a set of three common (*nodA*, *nodB*, *nodC*) genes and various other genes which encode the enzymes responsible for the synthesis and decoration of lipochitooligosaccharide molecules known as Nod factors. Nod factors differ among rhizobial species/strains and, once released by the bacteria, bind receptor proteins on the host plant, allowing a species-specific recognition, prior to the formation of the symbiotic nodule [12]. Diverse flavonoids may act as either an agonist or an antagonist of the same NodD protein [116], and different NodD proteins may differ in the specific agonist and antagonist. This extreme selectivity may explain part of the variable range of the transcriptional response to the same flavonoid (e.g., luteolin) by different strains of rhizobia [74]. However, flavonoids affect functions other than solely *nod* gene transcription. For instance, luteolin was shown to modulate the expression of motility genes in the *S. meliloti* 1021 strain [74]. In the same work, the presence of G × G interactions between three varieties of the host legume *M. sativa* and three rhizobia strains of *S. meliloti* was tested as the transcriptomic response of bacterial strains to root exudates. Interestingly, root exudates from the three plant varieties influenced a core set of functional gene categories in all three bacterial strains, and most of them were related to protein expression and modification, carbohydrate transport and metabolism and energy production and conservation [74]. Recent studies showed that luteolin can also modulate various substrate utilization pathways, as well as eliciting resistance toward stressors, including several antibiotics, toxic ions, respiration inhibitors, membrane damagers, DNA intercalants and other antimicrobial agents [117]. In other studies, it has been demonstrated that flavonoids can affect rhizobial chemotaxis by acting as the main attractant molecules prior to the initial signaling stage [118], and they can influence the production of proteins released in the soil from rhizobia via the type I and III secretion systems and the production of polysaccharides localized on the bacterial surface [119].

## 6. Role of the Rhizobial Mobile Genome in the Perception of the Plant

Several rhizobial species harbor a multipartite genome, i.e., a genome with a divided structure in several replicons, namely, a chromosome and various chromids, megaplasmids and plasmids [120]. Previous works and reviews have discussed the advantages of having a multipartite genome structure, such as maintaining a fast duplication rate while increasing the genome size and functional organization [78,120]. This genome organization is relatively frequent in bacterial species which interact with hosts and may colonize different environments, such as rhizobia. In rhizobia with a multipartite genome structure (such as those belonging to the genera *Sinorhizobium* and *Rhizobium*), genes encoding functions related to symbiosis and nitrogen fixation are mostly located on megaplasmids [121,122,123]. Such megaplasmids have clear signatures (such as the GC% content, codon usage and insertion sequences, IS) of alien origin with respect to the chromosome and likely spread relatively recently through horizontal gene transfer in rhizobia, giving rise to the evolution of the symbiotic phenotype [79,124]. Symbiotic megaplasmids represent classical examples of large mobile genetic elements (MGEs), providing novel and complex traits (i.e., requiring many genes), such as interactions with eukaryotic hosts [125]. In the rhizobial strain *S. meliloti* 1021, the symbiotic megaplasmid (pSymA ~1.4 Mb in size) represents nearly 21% of the genome and carries the genes critical for the symbiosis process such as *nod*, *nif* and *fix* [126]. Genetic experiments with mutants lacking the pSymA megaplasmid have clearly shown that this element is dispensable, but essential for symbiosis. Strains lacking pSymA are still able to thrive in the soil and colonize the rhizosphere but cannot allow the formation of symbiotic nodules [78,127]. Since pSymA contains genes required for plant recognition (e.g., *nod* genes), shuffling of the pSymA megaplasmid led to changes in the symbiotic phenotype [52]. Concerning plant signal recognition in particular, *nod* gene diversity can determine large variation in the host range. The term “symbiovar”, mentioned above, was introduced in the nomenclature of rhizobia aiming to reflect such an adaptation to different host species by the strains of the same rhizobial species [60]. This term emphasizes the spreading by horizontal gene transfer (HGT) of symbiotic plasmids and symbiotic-related genes. In this context, it should be noted that symbiotic megaplasmids often contain a number of mobile genetic elements, such as IS and mobile introns, which determine large structural variation [79]. Symbiotic megaplasmids harbor genes other than *nod*, *nif* and *fix* that are relevant in the interaction with the host plant and can provide different outcomes of symbiotic phenotypes. Among the most discussed is the gene encoding the *acdS* gene, which is claimed to be relevant for scavenging the ethylene precursor ACC, allowing a local decrease in ethylene and consequently nodule senescence [128]. However, the role of *acdS* is controversial, since the same gene of *S. meliloti* also appears to be expressed in the rhizosphere of non-host plants [90]. Other functions, possibly related to nitrogen metabolism, but also potentially involved in resistance to harsh environments, are located on pSymA, such as the gene encoding the nickel/proton antiporter *nreB* [129]. Interestingly, both *acdS* and *nreB* display evidence of HGT, uncoupled from the pSymA phylogeny, emphasizing the relevance of symbiotic megaplasmids as hot spots for the acquisition and spread of the rhizobial mobilome.

**Figure 2:**
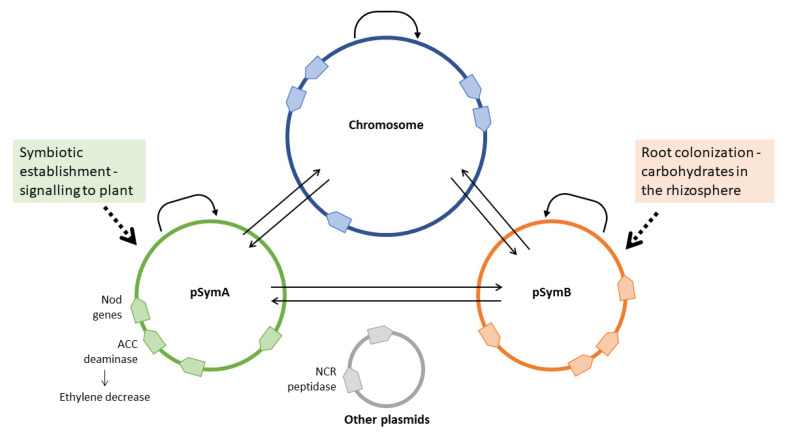
Partition and interaction among replicons in host genotype-specific interaction. The example reported refers to the *Sinorhizobium meliloti* genome, composed of a chromosome, a chromid (pSymB), a megaplasmid (pSymA) and often smaller accessory plasmids. Arrows connecting replicons are based on gene interaction data from [74,130,131].

Though the symbiotic megaplasmids, such as pSymA of *S. meliloti* 1021, confer the ability to become a symbiont, they are not simple “plug-and-play” modules, requiring a regulatory and metabolic connection to the other resident replicons of the genome (in *S. meliloti* 1021, the chromosome and the pSymB chromid) (Figure 2). Simulations of regulatory networks predicted the existence of several cross-replicon interactions [130], indicating that symbiotic megaplasmids need to be wired to the rhizobial genome. Transposon sequencing (Tn-Seq) experiments carried out on *S. meliloti* strains lacking pSymA and pSymB indicated that almost 10% of the chromosomal genes interact with genes located on the symbiotic megaplasmid pSymA and the pSymB chromid [131]. Concerning pSymB, several functions related to the exploitation of the rhizosphere, such as transport of carbohydrates, are present, suggesting a role in the differential exploitation of plant genotype-specific carbon sources in the rhizosphere [80,121]. In line with this hypothesis, transcriptome analysis of *S. meliloti* strains incubated with root exudates from different alfalfa varieties showed that pSymB stimulons (the set of differentially expressed genes after stimulation with root exudates) are highly variable either among strains or among conditions [74]. Among strains, stimulons ranged from 17% to 24% of the total differentially expressed genes, and among conditions (root exudates from different plant varieties and luteolin as a model flavonoid for *nod* gene induction), they ranged from 13% to 35% in the same strain [74], indicating that pSymB-harbored genes play a relevant role in the genotypic interaction with host plants.

Clearly, megaplasmids containing symbiosis genes, but also other plasmids (such as small accessory plasmids which may harbor NCR peptidases) and chromids, can be transferred to other rhizobia, promoting population evolution and the emergence of strains with a novel host genotype preference. Plasmid transfer is often controlled by quorum sensing, with high bacterial cell densities inducing transfer [132,133]. High cell densities could hardly be present in bulk soil, while they can likely be achieved in the rhizosphere, suggesting a role of the host in influencing the spread of symbiosis genes, similar to *Agrobacterium tumefaciens* pTi plasmid transfer [134]. However, though genetic dissection of conjugation genes has been performed, few data are available on the environmental signals and context of the symbiotic plasmid conjugal transfer [135,136,137,138]. Besides plasmids, within the rhizobia genome, symbiotic regions such as integrative and conjugative elements (ICEs [139]), determining host interaction and subjected to HGT, are present. Interestingly, an in vitro experiment showed that certain plant compounds inducing the nodulation process also improve the transfer of symbiosis islands in *Azorhizobium caulinodans* [140]. Here, the symbiosis island of 87.6 kb is an ICE capable of transferring to a site-specific gly-tRNA gene of other rhizobial species, expanding their host range. An AhaR transcriptional regulatory protein of the LysR family, located in the ICE, triggers the HGT process in response to plant flavonoids, which also induces the expression of nodulation genes through NodD. We can speculate that symbiotic megaplasmid transfer is controlled by similar environmental signals related to the rhizosphere or endosphere of host plants. Experiments clarifying these signals are needed to better understand the ecological context of symbiotic determinants spreading in the rhizobial populations.

## 7. A Cross-Talk in the Rhizomicrobiota

The more we delve deep into the complexity of symbiotic interactions, the more we discover that the overall biota surrounding plant roots has a relevance in the establishment of the success of symbiosis and of the phenotypes (i.e., performances) of both partners. It is becoming clear that many more signaling molecules than those initially discovered are present, including VOCs. These molecules play a role in both intermicrobial and interkingdom interactions. The acknowledgement of their role in microbe–microbe interactions is quite recent. Some of them exert antimicrobial activity, while others alter cell motility or induce biofilm formation, but little is known about the molecular mechanisms of these responses. On the other hand, their role in the interaction with plants has been known for almost two decades, and their emission improves plant growth by stimulating root development, chlorophyll production and the uptake of essential elements such as iron. They are also involved in plant tolerance to abiotic and biotic stresses, through antibiotic activity and activation of plant immunity [101].

**Figure 3:**
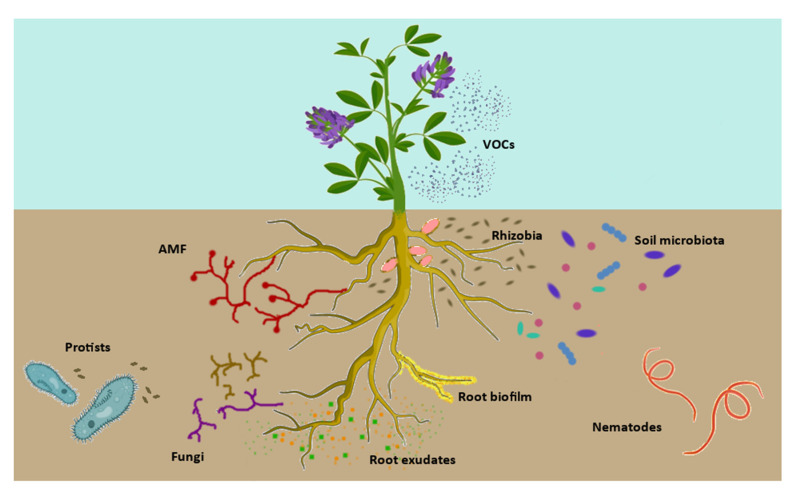
Cross-talk in the legume rhizosphere. Within- and among-kingdom interactions giving rise to modulation of symbiotic establishment are depicted, spanning from protists’ role as grazers of rhizobia to fungi, soil microbiota and nematodes which are attracted toward roots and may transport rhizobia.

Works on *Arabidopsis thaliana* have nicely disclosed the many actors and levels of interaction in the plant–microbe system [141,142]. Roots of a healthy plant host a variety of microorganisms belonging to different kingdoms, such as bacteria, archaea, protists and fungi (Figure 3). Bacterial communities are taxonomically structured, and their assembly is driven by factors such as soil type, plant compartment, host genotype/species and plant immune system. Fungal communities, in contrast, seem to be more influenced by their biogeographical distribution. Regarding protists, little information is available about community profiles. Among members of the plant-associated microbiota, two types of interactions can be identified: cooperation and competition. Microorganisms employ several cooperative mechanisms, such as nutritional interdependencies, biofilm formation, molecular communication through quorum sensing, enhanced dispersal and bacterial endosymbiosis in fungi, in order to ensure their persistence within the plant holobiont. Competitive mechanisms include resource competition through rapid utilization of limiting resources and secretion of siderophores, contact-dependent competition through the bacterial type VI and type III secretion systems, secretion of antibacterial and antifungal compounds and emission of VOCs produced by bacteria and predation [141]. For the predation aspect, we do not know if and how host legume plants perceive VOC signals from rhizobia and if, in turn, a plant which is forming nitrogen-fixing nodules emits VOCs to interact with microbes, other plants or pollinating insects. Setting up in vitro and in vivo experiments where VOCs are measured and identified using highly sensitive techniques such as proton transfer reaction-time of flight-mass spectrometry (PTR-TOF-MS) could allow shedding light on this still unknown side of rhizobia–host interaction.

For legumes, several clues on the interaction between rhizobia and fungi, especially arbuscular mycorrhizal fungi, are present (for a recent review, see [143]). Arbuscular mycorrhizal fungi (AMF) penetrate the cortical cells of the roots and ensure nutritional uptake to the plant. Mycorrhiza formation is guided by a complex signal exchange between the host plant and the AM fungus [144]. Strigolactone secreted by the plant induces spore germination, hyphal branching and hyphal growth toward the roots. This leads to the synthesis of short-chain chitin oligomers (CO4 and CO5), structurally similar to the rhizobial Nod factors. Recently, the receptors discriminating AMF and rhizobial signaling have been discovered and characterized [145]. Indeed, it is clear that AMF–plant symbiosis predates rhizobial–legume symbiosis, the latter being evolved from a previously settled molecular dialog. The first observation of AMF effects on nodulation and plant growth dates back almost fifty years [145]. Later on, many other beneficial effects, such as enhancement of symbiotic nitrogen fixation in drought soils, and the increase in the number and dry weight of nodules, have been observed [145]. In the establishment of the tripartite symbiosis among legumes, AMF and rhizobia, one study [146] highlighted the importance of specific flavonoids such as daidzein, genistein and coumestrol which act as signal molecules, playing a key role in the early stages of the interaction. This tripartite interaction represents a multiple mutualism where a host interacts with two mutualistic species. A recent study [147] showed that mycorrhizal fungi have a strong impact on fitness alignment between *M. truncatula* and rhizobia; in fact, in the presence of mycorrhizal fungi, rhizobia and plant fitness was more positively aligned and the strength of selection of a host trait (AMF branch number) doubled compared to plants inoculated with rhizobia alone.

Further dissection of the rhizospheric cross-talk is therefore crucial if we want to better understand the role of microbial networks in holobiont fitness, in view of agronomic applications [143]. Here, as mentioned before, a systems biology approach and more realistic experimental setups can help to overcome the symbiont- or host-centric single visions and clarify the effects of such symbioses on all partners [141,147].

## 8. Future Directions

A large array of molecular signals is exchanged between rhizobia and the host legume plant; some of them are crucial for the symbiosis to take place (such as flavonoids and Nod factors), while others are modifiers of the interaction. The latter have great importance in competition in the microbiota, in genotype-specific perception of host plants by the rhizobium and in the ability of the plant to maximize symbiont performances (i.e., nitrogen fixation and metabolic exchange) [143]. Moreover, the fungal component of the microbiota is stirring increased attention. Representing more than 70% of all land plants, legumes are able to directly interact with arbuscular mycorrhizal fungi (AMF), and direct molecular dialog is present between AMF and rhizobia [148]. However, we still do not know the molecular interactions rhizobia have with other key components of the soil biota, such as fungi other than AMF which are relevant components of bioinoculants used in agriculture (e.g., *Trichoderma*), or protists and nematodes, which can have an effect on the rhizobia population size (e.g., through grazing on rhizobia), but also the mobility of rhizobia toward the roots (e.g., nematode-carrying rhizobia being attracted by plant-released volatiles) [149].

A relatively simple problem, namely, rhizobium–plant symbiosis, is becoming very intricate, with many more genetic modifiers of the symbiosis than initially thought residing in the genomic variability of the partners, but also in a partnership which is not between two partners—the plant and the rhizobium—but with the overall soil biota. The possibility to exploit strains and genotypes for designing tailored bioinoculants, able to increase crop yield, must consider such intricate interactions. Consequently, we need to develop model systems, such as novel in vitro experimental setups and data interpretative models, to predict the outcomes from lab-scale analyses and trials to open field applications [150]. Systems biology approaches have proved to be powerful in finding key parameters in complex biological phenomena and have already shown their ability in modeling rhizobia symbiotic interaction [37,151,152]. Ecological modeling of plant–rhizobium–soil biota interactions [153], coupled with molecular data coming from genome analyses (see, for instance, [154] concerning human–microbe interaction), should be prioritized in order to find predictors (especially in the rhizobia genomes) of the goodness of the symbiotic phenotype in nature (and field conditions).
